# Engineering T Cell Function Using Chimeric Antigen Receptors Identified Using a DNA Library Approach

**DOI:** 10.1371/journal.pone.0063037

**Published:** 2013-05-07

**Authors:** Connie P. M. Duong, Jennifer A. Westwood, Carmen S. M. Yong, Amanda Murphy, Christel Devaud, Liza B. John, Phillip K. Darcy, Michael H. Kershaw

**Affiliations:** 1 Cancer Immunology Research Program, Sir Peter MacCallum Department of Oncology, University of Melbourne, Parkville, Victoria, Australia; 2 Department of Pathology, University of Melbourne, Parkville, Victoria, Australia; 3 Department of Immunology, Monash University, Prahran, Victoria, Australia; Carl-Gustav Carus Technical University-Dresden, Germany

## Abstract

Genetic engineering of cellular function holds much promise for the treatment of a variety of diseases including gene deficiencies and cancer. However, engineering the full complement of cellular functions can be a daunting genetic exercise since many molecular triggers need to be activated to achieve complete function. In the case of T cells, genes encoding chimeric antigen receptors (CARs) covalently linking antibodies to cytoplasmic signaling domains can trigger some, but not all, cellular functions against cancer cells. To date, relatively few CAR formats have been investigated using a candidate molecule approach, in which rationally chosen molecules were trialed one by one. Therefore, to expedite this arduous process we developed an innovative screening method to screen many thousands of CAR formats to identify genes able to enhance the anticancer ability of T cells. We used a directional in-frame library of randomly assembled signaling domains in a CAR specific for the tumor associated antigen erbB2. Several new and original CARs were identified, one of which had an enhanced ability to lyse cancer cells and inhibit tumor growth in mice. This study highlights novel technology that could be used to screen a variety of molecules for their capacity to induce diverse functions in cells.

## Introduction

Genes encoding chimeric antigen receptors (CARs) can be used to genetically modify T cells, which can be used in adoptive immunotherapy regimens to treat cancer. Anti-cancer CARs are generally composed of a single chain antibody variable fragment (scFv), specific for a tumor associated antigen, linked via a flexible hinge region through a transmembrane domain to T cell signaling domains. Existing T cell signaling molecules employed in pre-clinical and clinical studies include CD28, CD137 (4-1BB) and CD3ζ [1], [2], [3]. In these previous studies, the identification of functional CARs involved a candidate approach where signaling motifs were chosen in an intuitive manner and assembled in various orders together with a range of hinge and transmembrane domains [4]. While the candidate approach to CAR construction has yielded gene constructs with the ability to mediate anti-tumor responses in mice and humans, only a small proportion of the total complement of T cell signaling molecules has been investigated for activity in these systems [5], [6].

Ideally, T cells triggered through anti-tumor CARs would respond as strongly as naturally occurring T cells against foreign antigens such as virus. Virus-specific T cells can secrete large amounts of cytokines and mediate significant cytotoxicity at low effector to target ratios [7], [8]. In addition, a natural T cell response leads to enormous expansion of T cell populations together with the ability to traffic effectively to sites of disease and the capacity to establish populations of memory T cells able to persist long term. Current CAR-mediated T cell responses do not realize the full potential of T cell activation, which prompted us to investigate whether cytoplasmic domains of CARs containing further combinations of a range of signaling and adaptor molecules could lead to improvements in T cell activation.

T cell activation is a complex series of molecular events involving assembly of a variety of molecular species into a supramolecular activation cluster (SMAC) [9]. The SMAC is composed of primary activation molecules together with molecules whose roles include costimulation, adhesion and linker capacities. Examples of these molecules include activating components of the CD3 complex (including zeta, ζ), the costimulator CD28, and linker for activation of T cells (LAT) [10]. SMAC assembly is mediated by engagement of receptors on T cells with ligands displayed on target cells, which results in a localized molecular environment conducive to triggering of downstream signaling cascades leading to gene activation and production of biological mediators such as cytokines. While the natural process of T cell activation involves clustering of many different molecules mediated by a variety of ligands, it is possible to achieve clustering by linking cytoplasmic domains of several receptors in a continuous polypeptide chain to a single extracellular receptor to form a CAR [11]. Indeed, this strategy has been used to trigger T cell activation in response to ligation of CARs by cancer antigens [12], [13].

However, since assembling CARs using a candidate approach is extremely labor intensive involving long time periods before each CAR can be assessed, we devised a new strategy of CAR assembly. This strategy involved cloning a range of individual signaling and adaptor molecules in cassette form and ligating them in random order and number into the cytoplasmic domain of a retroviral vector encoding a CAR specific for the tumor-associated antigen erbB2 [14] to form a DNA library of CARs. The DNA library was screened for CARs able to induce Jurkat T cells to respond against erbB2, and promising CARs were validated in primary human T cells against cancer cells *in vitro* and *in vivo*.

## Results

### The CAR Library is Diverse in Size and Composition

To generate a DNA library of randomly assembled CARs we first flanked the DNA encoding individual signaling molecules with the sequence for the restriction enzyme SfiI that enables directional cloning of multiple signaling cassettes. PCR primers were designed to ensure in-frame ligation of cassettes. Together, these directional and in-frame strategies eliminated nonsense, reverse and prematurely terminated CARs, reducing library size by over 1,000-fold allowing us to focus on CARs with full length cytoplasmic domains. This strategy was key to building a library of multi-in-frame signaling molecules.

**Figure 1:**
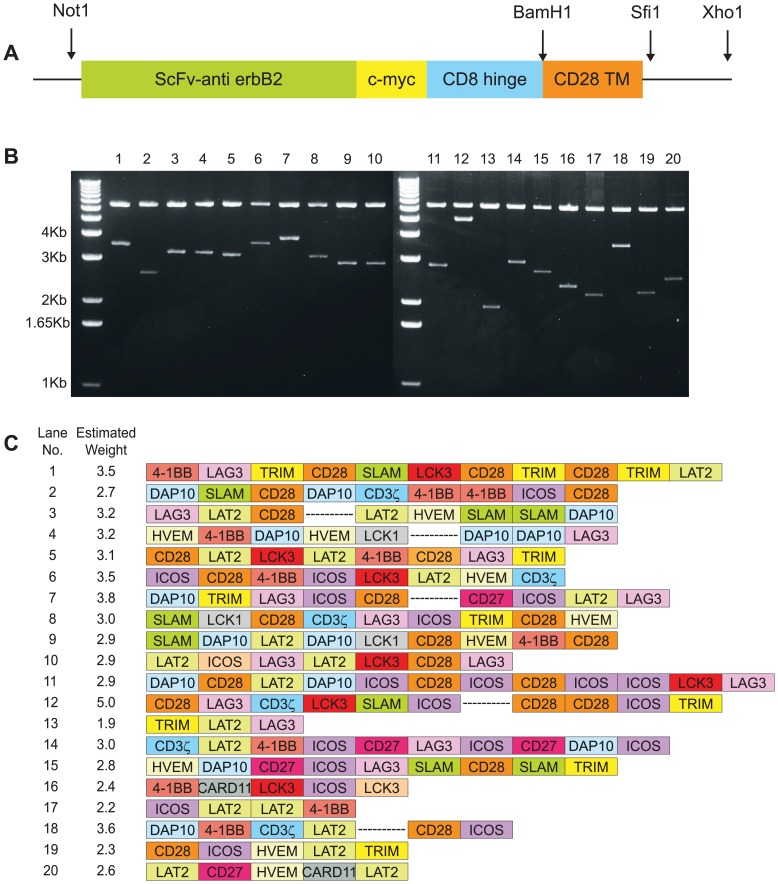
The chimeric cDNA library is diverse in size and composition. (**a**) The genetic construct is depicted encoding extracellular single chain anti-erbB2 linked via a c-myc tag and the hinge region from CD8 to the transmembrane domain of CD28. DNA encoding various signaling molecules was randomly ligated into the SfiI site. (**b**) Plasmids of 20 representative clones were digested with NotI and XhoI and analyzed by agarose gel electrophoresis. (**c**) DNA from the 20 clones was sequenced and represented as shown (not to scale). Corresponding clone numbers and weights from panel (b) are indicated. Break indicates unsequenced portions.

Fourteen signaling molecules (**Table 1**) were ligated in random number and order into the SfiI site of the pSAMEN-anti-erbB2 plasmid (**Fig. 1a**). The molecules were chosen as representative of primary stimulators, costimulators and adaptors, and although more signaling molecules are present in T cells, the number was kept at 14 for this initial proof of principle study. This generated a library of tumor targeting receptors, which was used to transform *E. coli*. We first investigated the diversity of the library using restriction enzyme digests of individual clones from *E. coli*. Using NotI and XhoI that cleave 5′ to the extracellular portion and 3′ to the cytoplasmic portion of the CAR respectively, a diverse range of sizes of molecules were revealed on agarose gel (**Fig. 1b**). The sizes of CARs varied from 1.1 kb, the size of the empty vector with no cytoplasmic inserts, to >4 kb, indicating a diverse variety of inserts.

**Table 1:** List of signaling and adaptor molecules.

Abbreviated/Alternate name	Molecule	Amino acids	Length (bp)	Accession number
CD137, 4-1BB	Tumor necrosis factor receptor superfamily, member (TNFRSF) 9	209–255	141	NM_001561
CARD11	Caspase recruitment domain family, member 11	26–113	261	NM_032415
CD27	Cluster of Differentiation 27	214–260	141	NM_001242
CD28	Cluster of Differentiation 28	180–220	123	NM_006139
DAP10	DNAX-activation protein 10	73–93	63	AF285447
HVEM	Herpesvirus entry mediator, TNFRSF14	226–283	174	NM_003820
ICOS	Inducible T-cell co-stimulator	165–199	105	NM_012092
LAG3	Lymphocyte activation gene 3	475–525	147	NM_002286
SLAMF1	Signaling lymphocytic activation molecule familymember 1	259–286	84	NM_003037
TRIM	T-cell receptor interacting molecule	28–100	219	AJ224878
CD247, CD3ζ	Cluster of Differentiation 247, zeta chain of CD3	52–100	147	NM_198053
Lck 1	Lymphocyte-specific protein tyrosine kinase	1–111	333	X13529
Lck 3	Lymphocyte-specific protein tyrosine kinase	212–311	300	X13529
LAT	Linker for activation of T cells	119–233	345	AF036905

Amino acids are numbered according to the accession number given. The length of each cDNA does not include the flanking SfiI sites.

DNA sequencing revealed integration of all of the individual cytoplasmic domains into the vector (**Fig. 1c**). In addition, a variable number and random order of inserts was observed. Thus, we achieved our aim of generating a diverse library of CAR containing multiple signaling domains. This plasmid library was used to create a retroviral producer cell line, PG13 [15], yielding a retroviral vector library pseudotyped with the gibbon ape leukemia viral envelope for transduction of human T cells.

### DNA Library Screening in Jurkat Cells Reveals Novel CAR Formats

**Figure 2:**
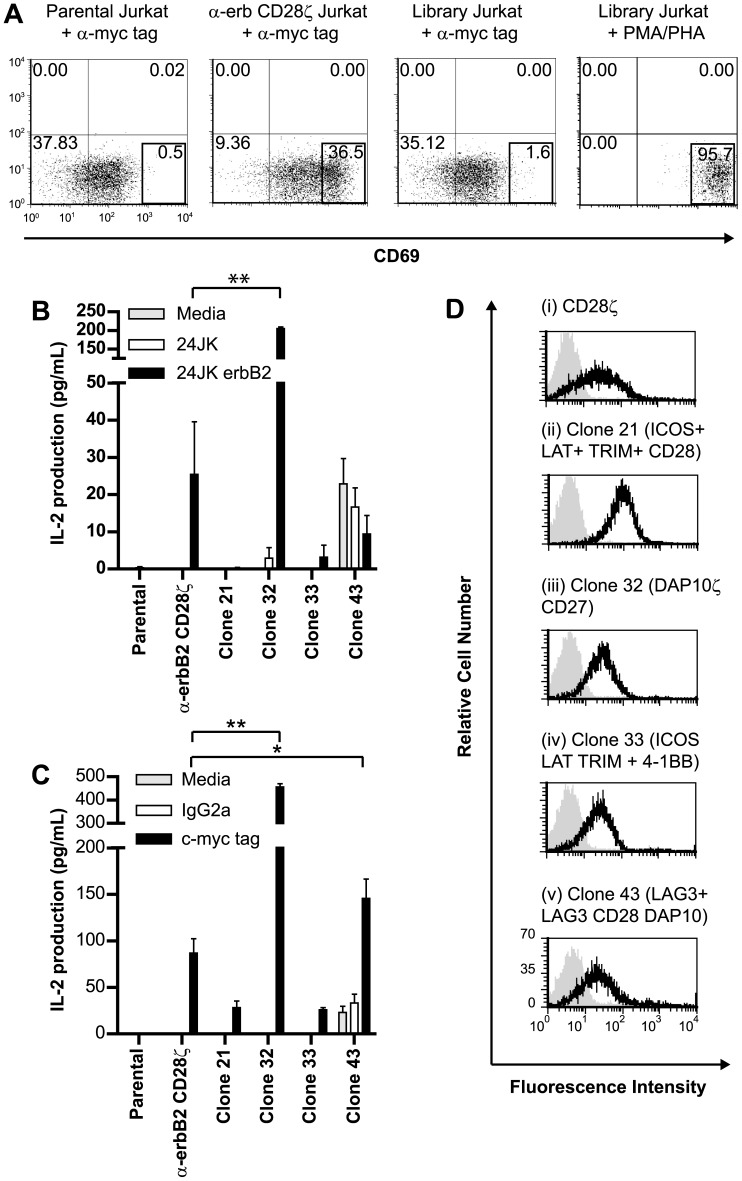
Novel chimeric receptors can enable Jurkat cells to respond following receptor ligation. (**a**) Populations of the human T cell line Jurkat (as listed) were incubated with anti-c-myc antibody and the expression of the activation marker CD69 determined. The boxed region represents cells with significant upregulation of CD69. Cells from the library-transduced population were subjected to fluorescence activated cell sorting and cells within the region cloned. CD69 upregulation in response to a positive control, phorbol myristate acetate (PMA) and phytohemagglutinin (PHA) is also depicted, together with upregulation in cells expressing a known active CAR, αerbB2CD28ζ. (**b**) Four clones were screened for their ability to secrete IL-2 in response to CAR ligation by erbB2 antigen, compared to the known positive control αerbB2CD28ζ. (**c**) IL-2 secretion from clones following incubation with anti-c-myc antibody (3 experiments pooled). (**d**) Expression of CARs in Jurkat clones was determined by flow cytometry following staining with anti-c-myc antibody. Four clones and the positive control Jurkat-erbB2CD28ζ are listed. (Error bars = SEM). *p<0.05, **p<0.005.

We anticipated that the CAR library would contain many non-functional CAR, since only relatively rare events would place stimulatory, costimulatory and/or adaptor molecules of suitable activity in the correct order to trigger T cell function. We wished to select highly functional clones that we could compare with the same anti-erbB2 scFv with our best signaling chain (anti-erbB2-CD28-CD3ζ). We could have screened the library directly into primary T cells, but these are varied and heterogenous and comparing receptors among clones from this population would have been difficult. We therefore screened the library of receptors for their ability to activate the human T cell line, Jurkat, in which all cells are genetically similar and so comparisons could be made between different CARs transduced into this line. After transducing the bulk population of Jurkat cells using the retroviral library, screening was performed by incubating the cell line in culture dishes coated with an antibody specific for the c-myc tag present in the extracellular domain of each CAR (**Fig. 1a**). In this way, we anticipated identifying CAR formats that could respond to molecular crosslinking producing synapses reminiscent of normal T cell signaling processes. Jurkat cells do not secrete all the repertoire of cytokines as naïve T cells, for instance they do not secrete IFNγ, but they do upregulate CD69 expression which has previously been determined to be a marker of activation [16], [17], and they secrete IL-2, so these two screening options were available for selecting functional clones from the Jurkat cells transduced with our library of CARs. Therefore, following incubation with anti-c-myc Ab, Jurkat cells were stained for CD69 expression. Flow cytometric analysis was then performed, and cells with CD69 upregulated above that of parental (non-transduced) Jurkat cells were isolated and cloned into 96-well culture plates to yield a total of 147 clones (**Fig. 2a**). These clones were then individually re-screened for CD69 upregulation in response to CAR ligation, and 39 were confirmed as responders. The 21 highest CD69-expressors were screened for IL-2 secretion in response to anti-c-myc antibody, and 11 were demonstrated to produce IL-2. The 4 highest IL-2 secretor clones identified in this initial screen were then selected for further analysis and cultured with anti-c-myc antibody or the sarcoma cell line 24JK transfected to express erbB2 or controls, and IL-2 secretion determined. In this screen, some clones produced levels of IL-2 comparable to, or better than our previously determined functionally optimal CAR, αerbB2-CD28ζ (**Fig. 2b and 2c**). These clones were also determined to express CARs to varying degrees (**Fig. 2d**).

To determine the composition of active CARs, genomic DNA was isolated from functional Jurkat clones and PCR used to clone and sequence CAR cytoplasmic domains. A variety of cytoplasmic domains incorporating a diversity of signaling molecules was found (**Figure 2d**). As determined in the plasmid library, the Jurkat cytoplasmic domains contained various numbers of signaling molecules in random order, although the cytoplasmic domains were generally smaller than those in the plasmid library, probably due to the known preference for incorporation of smaller RNA species into retroviral vectors. Some Jurkat cells contained a single CAR, whereas others contained several individual CARs. Based on our observations of up to 4 signaling domains in individual Jurkat cells, we estimated the number of possible CAR combinations in Jurkat to be >30,000 (14^4^). Some CARs contained both primary activation signaling domains (TCR-ζ) and costimulatory domains (e.g. CD27, CD28), as expected from candidate approaches to CAR design. However, some highly novel CAR containing less intuitive combinations of adaptor and costimulatory cytoplasmic domains were also found. Interestingly, this library approach re-isolated αerbB2-CD28-ζ as a functional CAR, thereby verifying the capacity of the approach to identify CAR previously discovered using candidate approaches [18].

**Figure 3:**
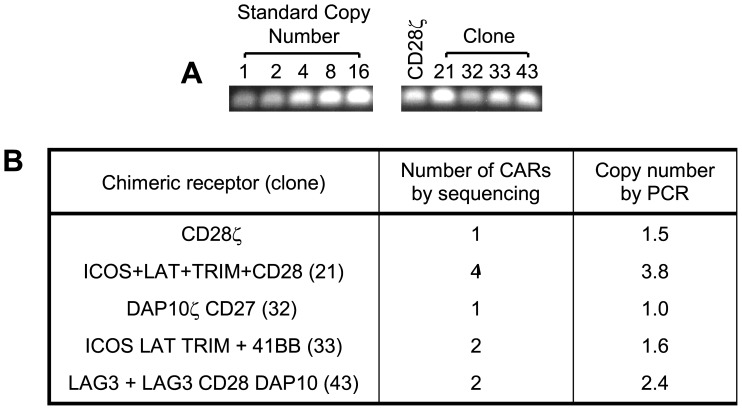
Copy number of chimeric receptors in Jurkat cell lines evaluated by PCR. The intensity of the PCR products from Jurkat cell lines expressing chimeric receptors was compared to standard copy number of parental Jurkat genomic DNA spiked with 1–16 copies of a CAR by (**a**) PCR and quantified using (**b**) Metamorph software. Shown is a representative experiment from 3 experiments performed.

Retroviral biology dictates that a single virus integrates one provirus into the host cell genome, but to gain further insight into the viral copy number in the Jurkat clones, and determine if our sequencing strategy had missed some inserts, we isolated genomic DNA and performed quantitative PCR as described in Materials and Methods. The copy number for Jurkat clone 32, bearing the DAP10ζCD27 CAR, was 1.0, indicating that was the only CAR present (**Figure 3**). Jurkats with the CD28ζ CAR had a copy number of 1.5, suggesting 1 to 2 viral copies per cell. Clone 21 had 3.8 virus copies (most likely 4), which agreed with the number found by sequencing, indicating we had not missed other integrants. Clones 33 and 43 that had 2 CARs each by sequencing, were found to have 1.6 and 2.4 viral copies by PCR (most likely 2), suggesting the sequenced CARs were the only ones present. Interestingly, we did not observe a correlation between viral copy number and the level of CAR expression by Jurkat clones (**Fig. 2d**).

### A Novel CAR Enables Human T Cells to Respond against Cancer Cells *in vitro*

**Figure 4:**
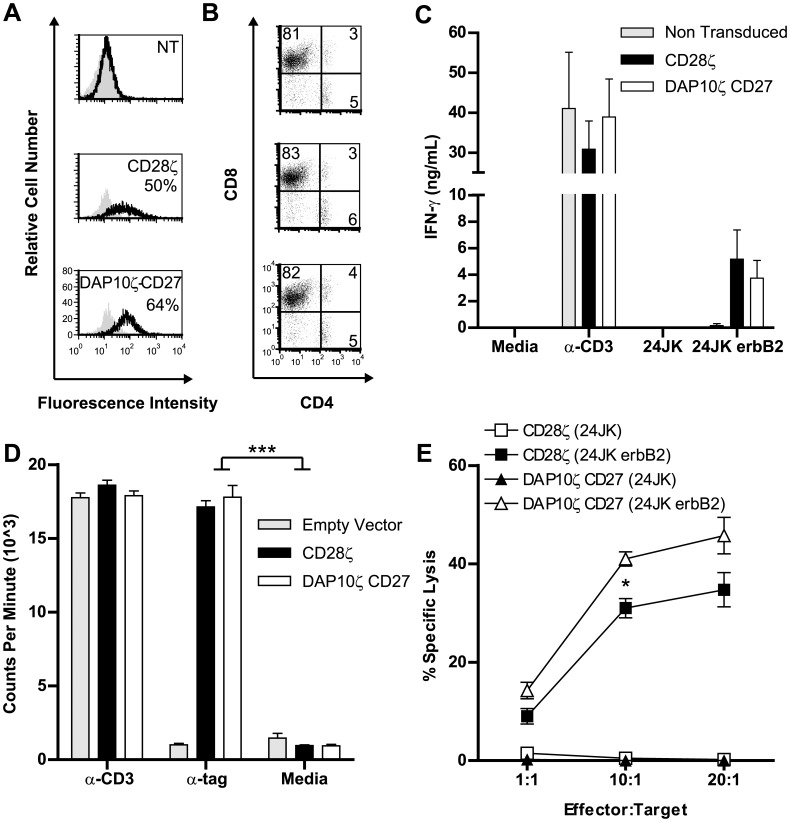
Human primary T cells bearing the αerbB2DAP10ζCD27 CAR respond against tumor cells. (**a**) Expression of CARs (αerbB2-CD28ζ: 57.99±12.66, αerbB2-DAP10ζCD27: 66.38±22.90. N = 3± SEM, p = 0.54) and (**b**) expression of CD4/CD8 in transduced human T cells was determined by flow cytometry (representative plot of 3 experiments). (**c**) The ability of T cells to secrete cytokines in response to CAR ligation was determined by ELISA after overnight incubation with tumor cells (3 experiments pooled). (**d**) The ability of CARs to mediate T cell proliferation was determined by incorporation of tritiated thymidine following incubation of T cells with anti-c-myc antibody. (Representative of 2 experiments), ***p<0.0005. (**e**) The antigen-specific cytolytic potential of CAR-expressing T cells was determined *in vitro* using a ^51^Cr release assay using target cells expressing erbB2 or not expressing erbB2. (4 experiments pooled), *p = 0.0147.

To validate and extend the findings from Jurkat, we expressed the αerbB2-DAP10ζCD27 CAR in human peripheral blood lymphocytes (PBL) and compared its function against our existing CAR αerbB2-CD28ζ. Expression levels of each CAR were found to be comparable, with no significant difference in mean fluorescence intensity (αerbB2-CD28ζ: 57.99±12.66, αerbB2-DAP10ζCD27: 66.38±22.90. N = 3± SEM, p = 0.54) (**Fig. 4a**). In addition, the composition of CD4^+^ and CD8^+^ T cell subsets was similar for T cells bearing either chimeric receptor (CD28ζ: CD4^+^14±4.9, CD8^+^74.7±4.6, αerbB2-DAP10ζCD27: CD4^+^15±6.1, CD8^+^75.3±4.4. N = 3± SEM_CD4_, p = 1, SEM_CD8_ p = 0.83) (**Fig. 4b**). Thus, any differences in function would not be due to variation in expression levels of CAR or inherent differences in subset composition.

Since human PBL can secrete a wider range of cytokines than Jurkat cells, we sought to extend the findings to another important cytokine, IFN-γ. Comparable levels of IFN-γ were secreted by both CARs in response to incubation with erbB2^+^ tumor cells (**Fig. 4c**). Since antigen-induced expansion of T cells is desirable in therapeutic application of CAR T cells against cancer, we determined the relative ability of the CD28ζ and DAP10ζCD27 CARs to stimulate proliferation of T cells. Proliferation was found to be similar in response to CAR ligation (**Fig. 4d**).

Given the importance of cytotoxicity in T cell function, we next sought to compare the ability of each CAR to lyse erbB2^+^ tumor cells. T cells bearing the DAP10ζCD27 CAR were significantly better at lysing 24JK-erbB2 cells than T cells bearing CD28ζ CAR (**Fig. 4e**). Taken together, these results demonstrated that a new library-isolated CAR could induce T cells to proliferate and secrete cytokines similarly to a previously identified CAR, but with enhanced cytotoxic ability. Other investigators have identified CARs containing 3 cytoplasmic domains with superior cytokine secreting abilities over CARs containing 2 domains using a candidate molecule approach, but enhancement of cytotoxicity is not generally observed [2], [19], [20].

### T Cells Expressing the αerbB2-DAP10ζCD27 CAR Inhibit Tumor Growth in Mice

**Figure 5:**
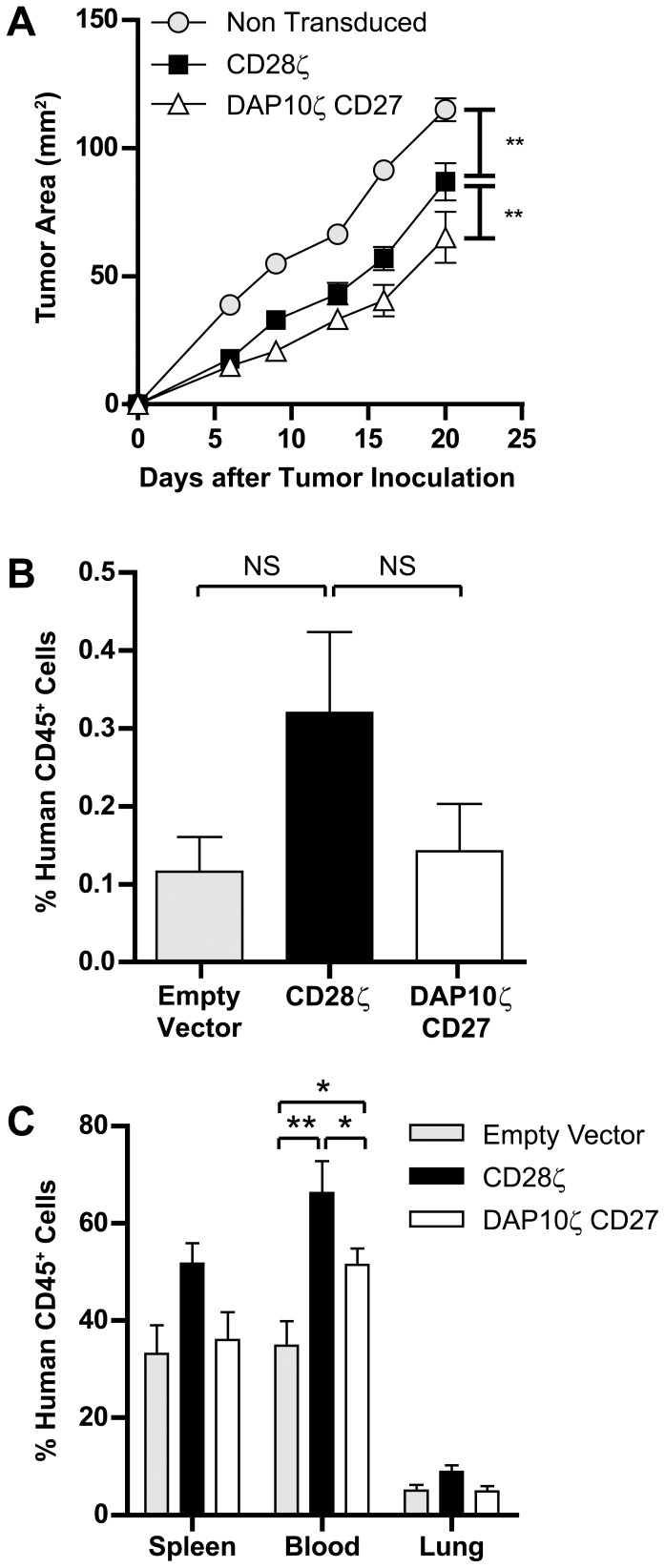
Human T cells bearing the αerbB2DAP10ζCD27 CAR inhibit tumor growth in mice. (**a**) CAR-expressing human T cells were injected on days 0, 1 and 2 intravenously into NOD-SCID mice injected subcutaneously on day 0 with 24JK-erbB2 sarcomas, and tumor growth monitored (9–10 mice/group). (**b,c**) Localization of T cells was determined on dissociated tumor and tissues on day 4 of tumor growth using flow cytometry (5 mice/group). (Error bars = SEM). *p<0.05, **p<0.005, NS = not significant.

In addition to comparisons of T cell function *in vitro*, their relative ability to function *in vivo* is also of interest, which may reveal differences in anti-tumor ability or their capacity to persist or accumulate in tumors. To determine this we injected the erbB2^+^ cell line 24JK-erbB2 subcutaneously in NOD-SCID mice and transferred CAR-bearing T cells or control T cells intravenously. T cells bearing the DAP10ζCD27 CAR inhibited tumor growth to a greater degree than T cells expressing the CD28ζ CAR (**Fig. 5a**). The relative persistence of CAR T cells was determined by flow cytometric analysis of spleens, lung, blood and tumor four days after tumor cell injection, with T cells injected intravenously on days 0, 1 and 2. Localization of T cells to tumors was found to be comparable for the two CAR T cell populations (**Fig. 5b**). Interestingly, the frequency of tumor infiltrating human lymphocytes was very low in all groups of mice, perhaps highlighting the importance of an increased T cell cytolytic capacity when only low numbers of T cells localize to tumors. T cell persistence in the lungs and spleen was observed to be similar for both CAR-bearing populations (**Fig. 5c**). However, CAR-bearing T cells were present at significantly higher frequencies in the blood compared to control vector transduced T cells, and T cells expressing CD28ζ were present at a higher frequency than T cells expressing DAP10ζCD27. These data demonstrated the ability of a CAR with a newly identified combination of signaling moieties to endow T cells with the ability to inhibit tumor growth in mice. These findings support the crucial role played by cytotoxic lymphocytes in eradicating tumors in numerous animal models of disease.

## Discussion

The cytoplasmic domain of the enhanced CAR contained DAP10, CD27 and ζ linked together sequentially. It is likely that ζ functioned as the primary activating molecule with costimulation from CD27, similar to that observed for CD28 [21]. Indeed, ζ and CD27 have been demonstrated to cooperate to mediate enhanced anti-tumor activity [22]. DAP10 has been previously shown to be important in mediating cytotoxicity [23], [24]. Thus, it seems likely that all three cytoplasmic domains participated in mediating the observed T cell function, although definitive insight into the contribution of each domain would require screening each cytoplasmic domain individually and in various combinations and orders, which was beyond the scope of this initial study that focused on the technological advance. The current study serves as proof of principle that a range of functional CAR can be identified using this technology of random library construction.

A key aspect of the technology described in this study is the directional in-frame nature of the molecular library produced. Without this feature, the library size would be several orders of magnitude greater, including largely non-functional molecular species, which would render screening extremely difficult. Even with this feature only a relatively small proportion of CARs were found to activate Jurkat cells. This is not entirely surprising since previous studies using the candidate approach have determined that not only is the composition of signaling domains important, but their order can also impact on function. Although cytotoxicity was the only significantly different function mediated by the DAP10CD27ζ receptor, we cannot rule out further differences in other functions such as secretion of other molecular species not specifically investigated in this study.

At present, it is not clear why T cells expressing the DAP10CD27ζ CAR tended to be present at lower frequency than those expressing the CD28ζ CAR in tumor-bearing mice, although it may reflect lower survival of T cells expressing the more lytic CAR over the relatively short period of 4 days. It would be interesting to determine the relative persistence of these T cells at later time points, although this would likely require switching models to using mouse T cells in a syngeneic model, since human T cells do not persist well in mice.

While this study utilized a selection of only 14 cytoplasmic domains randomly joined in-frame and directional to form a library of signaling molecules for screening T cell function, this technology could be used to screen a variety of other molecules for their capacity to induce diverse functions in a wide range of other cell types. Perhaps the most immediate use would be with cellular functions that are elicited through ligand-induced association of several different molecules with enzymic or adapter roles in signaling cascades. However, there is also the potential to enhance the function of individual molecules through random association of various intramolecular subdomains. Thus, it may be possible to design growth factor receptors or cytokines with enhanced multifactorial capabilities in order to utilize cellular function for a range of applications.

While the magnitude of improvement in CAR function in this preliminary proof of principle study is not great, it is important to realize that the entire CAR library was not screened and better CARs may be identified in future. Indeed, there was a disparity between the plasmid library size (>1×10^8^) and Jurkat library size (>3×10^4^), which was probably due to the limitations of retroviral vectors that tend to prefer shorter inserts, and therefore some very interesting longer constructs may have been missed using the current approach. In future we may be able to address this limitation using alternate vectors and transduction methods.

We used a MOI of up to two in our screening of the library in Jurkat cells, which may have contributed to the integration of multiple CARs in some Jurkat clones. Although this may help identify interesting associations between individual CARs, it makes interpretation of the results more complex, and therefore a multiplicity of infection of less than one would likely be a better strategy in order to decrease the possibility of integration of several CARs into the same cell.

In our study, even though the improvement to CAR function was modest, much of the importance of the work lies in the technological approach to identify promising new CAR formats. The strength of this technology is the ability to identify numerous novel chimeric molecules from a huge range of possible candidates, in a rapid manner compared to a conventional candidate approach. Further development of this approach, for example with other specificities and including additional signaling and adaptor molecules, could lead to rapid advances in our quest to harness various cell functions against cancer.

## Materials and Methods

### Cell Lines

The mouse (C57BL/6) sarcoma cell line 24JK (kindly provided by Dr. Patrick Hwu and Dr Nicholas Restifo, National Institute of Health, Bethesda, MD) [25], human T cell line, Jurkat (E6-1) (American Type Culture Collection) (ATCC) and retroviral vector packaging cell lines GP+E86, PA317 and PG13 (all from ATCC) and derivatives of these cell lines were maintained in complete medium consisting of RPMI (Gibco, Life Technologies, Grand Island, NY) with 10% heat-inactivated fetal calf serum (FCS) (MultiSer, Thermo Trace, Melbourne) and additives (2 mM glutamine (Gibco), 0.1 mM non-essential amino acids (NEAA) (Gibco), 1 mM sodium pyruvate (Gibco), 100 µg/mL streptomycin (Sigma-Aldrich, St Louis, MO) and 100 U/mL penicillin (Sigma-Aldrich) in a humidified incubator at 37°C with 5% CO_2_.

### Generation of CAR Signaling Library

The cDNA sequences of the cytoplasmic domains of 14 signaling molecules (**Table 1**) were amplified with primers incorporating a SfiI site on both the 5′ and 3′ ends. SfiI is a restriction enzyme that is unidirectional so that DNA must always ligate forwards. The signaling molecules were then subcloned into pCR®4-TOPO® vector plasmid (Life Technologies, Carlsbad, CA, USA) and verified by sequencing.

The signaling molecules were then cut with SfiI and randomly ligated together into the plasmid, pSAMEN encoding for anti-erbB2 scFv linked to a CD8 hinge and CD28 transmembrane followed by SfiI sites (**Fig. 1a**), lacking a cytoplasmic domain, to produce a plasmid library of chimeric antigen receptors (CARs). The plasmid library was enriched for heavy molecular species (containing multiple signaling motifs) after agarose gel electrophoresis. The pSAMEN CAR library was transfected into a mixture of GP+E86 and PA317 retroviral packaging lines to initiate a “ping-pong” process [26] to produce a high titer retroviral vector producer cell line (predominantly PA317 due to the slow growth rate of GP+E86). This cell line was selected in G418, and supernatant used to infect the PG13 packaging cell line [15] several times to generate a high titer retroviral vector pseudotyped in the Gibbon ape leukemia virus envelope protein.

### Generation of Jurkat Cell Line Expressing the Signaling Library

Jurkat cells were transduced with supernatant from the PG13 library cell line. One day prior to retroviral transduction, the PG13 retroviral producer cell line was seeded at 1×10^7^ in a 175 cm^2^ tissue culture flask in 15 mL of complete RPMI. Six-well tissue culture plates (Multiwell, Becton Dickinson, San Jose, CA) were coated with 30 µg RetroNectin (Takara, Japan) in 2 mL PBS, 24 hours prior to retroviral transduction. For transduction, 5 mL of retroviral supernatant (0.5×10^6^ CFU/mL) from the PG13 line was placed into RetroNectin-coated 6 well plates for 4 hours at 37°C. Following this incubation, 2.5×10^6^ Jurkat cells were resuspended in 5 mL of complete RPMI, and placed into retrovirus-coated wells. This transduction process was repeated the next day on fresh RetroNectin and virus-coated plates. The maximum MOI was therefore 2. Twenty four hours after the transduction process, 750 µg/mL of G418 (Gibco) was added. After seven days of G418 selection, cells were resuspended in fresh media and subjected to flow cytometry for chimeric receptor expression and phenotypic analysis and used for *in vitro* assays.

### Isolation of Clones with Highest Functional Signaling Chains

The Jurkat cell line expressing the signaling library was cultured overnight on tissue culture plates coated with an antibody specific for the c-myc tag incorporated into the extracellular domain of the CAR, which acted as a surrogate to erbB2 tumor antigen to ligate CAR. Cells were then stained and sorted by flow cytometry for upregulation of the CD69 activation marker and single-cell cloned into 96-well plates. Clones were subsequently screened for IL-2 secretion in response to overnight incubation with anti-c-myc antibody or 24JK-erbB2 target cells. The genomic DNA of the clones with the highest amount of antigen specific IL-2 secretion was isolated using a kit according to manufacturer’s instructions (QIAGEN, GmbH, Hilden, Germany). Genomic DNA was then used as a template for PCR cloning of the cytoplasmic domain of active CARs into the vector pCR®4-TOPO® to enable sequencing. DNA was isolated from 10 *E. coli* colonies and digested with EcoR1 and run on an agarose gel. When more than 1 band was present, DNA from 1–4 clones with representative DNA of each size was sequenced. In this way we sought to determine if multiple CARS were inserted in single Jurkat clones. However, it was still possible that screening 10 colonies may have missed additional inserts.

### Retroviral Transduction of Primary Human T Cells

Peripheral blood mononuclear cells (PBMCs) were obtained from buffy coats from healthy donors (Australian Red Cross Blood Service) approved by the Peter MacCallum Cancer Centre Human Research Ethics Committee (Project 01/14). PBMCs were isolated by Ficoll-Isopaque (GE Healthcare, NY) density centrifugation, and resuspended at 1×10^6^/mL in 50 mL of complete RPMI. Interleukin-2 (600 IU/mL) and α-CD3 monoclonal antibody (OKT3, Orthoclone, 30 ng/mL) were also included in the culture to induce proliferation of T cells. T cells were transduced to express CARs utilizing a similar protocol to Jurkat transduction, with the addition of a centrifugation step at 1000 g at room temperature for 60 min immediately after adding T cells to tissue culture plates.

### Flow Cytometry

The receptor expression on transduced human T cells was analyzed by flow cytometry. Cells were stained with Alexafluor-488-conjugated anti-c-myc antibody (9B11, Cell Signaling Technology, Beverly, MA). In order to determine the lymphocyte subset populations, cells were also stained with PE Cy7-anti-CD8 (Becton Dickinson) and APC-anti-CD4 (Becton Dickinson). Analysis of antigen expression on tumor cell lines and chimeric receptor expression and phenotypic analysis of human T cells was performed using a LSR II flow cytometer (Becton Dickinson) and FCS Express software (De Novo Software, City CA).

### Cytokine Production and Cytotoxicity Assays

Cytokine secretion by human transduced T cells was measured by enzyme linked immunosorbent assays (ELISA) following incubation with immobilized anti-c-myc antibody or antigen positive cells or controls. Transduced T cells (2×10^6^/mL) were cultured with 1×10^6^/mL target cells in complete RPMI. No exogenous IL-2 was added. Immobilized α-CD3 was used as a positive control. Following overnight incubation, supernatants were harvested and interferon γ (IFN-γ) and IL-2 secretion were determined by ELISA. The cytolytic capacity of transduced T cells was analyzed using a 4 hour ^51^Cr release assay.

### Quantitative PCR

PCR was used to evaluate the copy number in Jurkat cell lines expressing chimeric receptors. Genomic DNA (Qiagen) was isolated from cells and quantified (NanoDrop 2000, Thermo Scientific). Primers binding to the scFv were subsequently used to amplify the CAR DNA. Following agarose gel electropheresis, the intensity of bands was compared to copy number standards containing CAR-plasmid seeded into genomic DNA isolated from the Jurkat parental line. The intensity of PCR products was quantified using Metamorph software (Molecular Devices).

### Thymidine Incorporation Assay

Genetically modified human T cells were cultured in 96-well flat-bottom plates with the following conditions: media alone, plastic-immobilized anti-c-myc monoclonal antibody (1 µg/mL), or immobilized α-CD3 (OKT3, Orthoclone, 2**µg/mL) 2 days. The cultures were pulsed with [^3^H]thymidine (0.5 µCi/well) (Amersham, Aylesbury, UK) for the final 16 hr of the culture period and the incorporation of radioactivity was measured in a TRI-CARD 2100 TR liquid scintillation counter (Packard, Meriden, CT).

### Mouse Tumor Model

Ethics Statement: This study was carried out in strict accordance with the recommendations of the Victorian Bureau of Animal Welfare, Department of Primary Industries, and the National Health and Medical Research Council’s Australian code of practice for the care and use of animals for scientific purposes. The protocol was approved by the Peter MacCallum Cancer Centre Animal Experimentation Ethics Committee under Permit number E396. All efforts were made to minimize suffering.

Inbred non-obese diabetic severe combined immunodeficient (NOD-SCID) mice (Walter and Eliza Hall Institute, Melbourne) were irradiated at 2.5 Gy. Mice were subsequently injected subcutaneously with 1×10^6^ of the 24JK-erbB2 cell line. Mice were injected intravenously with 1×10^7^ primary human T cells transduced to express either anti-erbB2 CD28ζ or anti-erbB2 DAP10ζ CD27 or control T cells on days 0, 1 and 2. Following adoptive transfer of T cells, tumor growth was monitored using calipers along two perpendicular axes of the tumors. Mice for the survival experiment were culled when tumors reached 150 mm^2^ in size or at first signs of stress. Mice were culled on day 4 after tumor inoculation for investigating localization of T cells to various tissues.

### Statistical Analysis

Statistical significance in experiments comparing gene expression, cytokine production, cytotoxicity of transduced T cells and *in vivo* tumor growth was determined by two-way Analysis of Variance (ANOVA) in Graphpad Prism (Graphpad Software, San Diego, California).
